# Mapping of *Mcs30*, a New Mammary Carcinoma Susceptibility Quantitative Trait Locus (QTL30) on Rat Chromosome 12: Identification of *Fry* as a Candidate *Mcs* Gene

**DOI:** 10.1371/journal.pone.0070930

**Published:** 2013-09-02

**Authors:** Xuefeng Ren, Jessica C. Graham, Lichen Jing, Andrei M. Mikheev, Yuan Gao, Jenny Pan Lew, Hong Xie, Andrea S. Kim, Xiuling Shang, Cynthia Friedman, Graham Vail, Ming Zhu Fang, Yana Bromberg, Helmut Zarbl

**Affiliations:** 1 Department of Social and Preventive Medicine, the State University of New York, Buffalo, New York, United States of America; 2 Guangdong Medical Laboratory Animal Center, Foshan, Guangdong, China; 3 Department of Environmental and Occupational Medicine, Robert Wood Johnson Medical School, University of Medicine and Dentistry of New Jersey Piscataway, New Jersey, United States of America; 4 Joint Graduate Program in Toxicology. Rutgers, The State University of New Jersey University and the University of Medicine and Dentistry of New Jersey, Piscataway, New Jersey, United States of America; 5 NIEHS Center for Environmental Exposures and Disease, University of Medicine and Dentistry of New Jersey and Rutgers University, Piscataway, New Jersey, United States of America; 6 Environmental and Occupational Health Sciences Institute, University of Medicine and Dentistry of New Jersey and Rutgers University, Piscataway, New Jersey, United States of America; 7 Department of Biochemistry and Microbiology, Rutgers University, New Brunswick, New Jersey, United States of America; 8 Cancer Institute of New Jersey, New Brunswick, New Jersey, United States of America; 9 Fred Hutchinson Cancer Research Center (FHCRC), Seattle, Washington, United States of America; 10 NIEHS Center for Ecogenetics and Environmental Health, and the Department of Environmental and Occupational Health, University of Washington, Seattle, Washington, United States of America; Ohio State University Medical Center, United States of America

## Abstract

Rat strains differ dramatically in their susceptibility to mammary carcinogenesis. On the assumption that susceptibility genes are conserved across mammalian species and hence inform human carcinogenesis, numerous investigators have used genetic linkage studies in rats to identify genes responsible for differential susceptibility to carcinogenesis. Using a genetic backcross between the resistant Copenhagen (Cop) and susceptible Fischer 344 (F344) strains, we mapped a novel mammary carcinoma susceptibility (*Mcs30*) locus to the centromeric region on chromosome 12 (LOD score of ∼8.6 at the D12Rat59 marker). The *Mcs30* locus comprises approximately 12 Mbp on the long arm of rat RNO12 whose synteny is conserved on human chromosome 13q12 to 13q13. After analyzing numerous genes comprising this locus, we identified *Fry*, the rat ortholog of the furry gene of *Drosophila melanogaster,* as a candidate *Mcs* gene. We cloned and determined the complete nucleotide sequence of the 13 kbp *Fry* mRNA. Sequence analysis indicated that the *Fry* gene was highly conserved across evolution, with 90% similarity of the predicted amino acid sequence among eutherian mammals. Comparison of the *Fry* sequence in the Cop and F344 strains identified two non-synonymous single nucleotide polymorphisms (SNPs), one of which creates a putative, de novo phosphorylation site. Further analysis showed that the expression of the *Fry* gene is reduced in a majority of rat mammary tumors. Our results also suggested that FRY activity was reduced in human breast carcinoma cell lines as a result of reduced levels or mutation. This study is the first to identify the *Fry* gene as a candidate *Mcs* gene. Our data suggest that the SNPs within the *Fry* gene contribute to the genetic susceptibility of the F344 rat strain to mammary carcinogenesis. These results provide the foundation for analyzing the role of the human *FRY* gene in cancer susceptibility and progression.

## Introduction

Breast cancer remains the most prevalent cancer among US women, with an estimated 226,870 new cases and 39,510 deaths occurring in 2012 (National Cancer Institute SEER Cancer Statistics). Although a multitude of genetic alterations and molecular pathways have been implicated in the pathogenesis of breast cancer [1], [2], there remain significant gaps in knowledge regarding the biology and etiology, in particular, genetic susceptibility to this disease.

Rat strains vary widely in their genetic susceptibility to mammary carcinogenesis. On the assumption that tumor suppressors are conserved across mammalian species, investigators have carried out numerous genetic linkage studies in rats to identify genes responsible for this differential susceptibility [3], [4], [5], [6], [7], [8], [9], [10]. Prior studies in rats identified 29 strain-specific mammary carcinoma susceptibility (Mcs) loci [11], [12]. Several candidate *Mcs* genes, including *Mcs1b*, Mcs*5a1* and Mcs5c, were identified within several of these loci [6], [7], [8]. Identification and functional characterization of additional *Mcs* genes will enhance our understanding of the genetic basis for the differential susceptibility to mammary carcinogenesis.

Previous segregation analyses indicated that a genetic cross between the resistant Cop and the intermediately-sensitive F344 strain would involve a minimal number of genetic modifiers [13], [14], and hence could facilitate genetic analyses. We therefore performed genetic linkage analysis and interval mapping in [(F344 X Cop) F1 X F344] N2 backcross progeny to identify loci that conferred susceptibility to N-Methyl-N-Nitrosourea (NMU)-induced mammary carcinogenesis in the F344 rat. Using tumor number as the quantitative trait, we mapped a previously unreported susceptibility locus, *Mcs30* (QTL30), to rat chromosome 12 (LOD score ∼8.6). We identified and evaluated several candidate carcinoma susceptibility genes located within this locus. Comparative DNA sequence analysis, functional prediction, and analysis of FRY expression in rat mammary tumors and human breast cancer cell lines strongly indicate that *Fry*, the rat ortholog of Drosophila *furry* gene [15], encodes a *Mcs* gene within the susceptibility locus (QTL30) on rat chromosome 12 (RNO12).

## Materials and Methods

### Animals and Breeding Strategies

Ethics Statement: All animal protocols were reviewed and approved by the Institutional Animal Care and Use Committee of Fred Hutchinson Cancer Research Center (FHCRC). All experiments were performed in FHCRC vivarium, which is a fully accredited by AAALAC.

Copenhagen (Cop), Fischer344 (F344) and Brown Norway (BN) rats were purchased from Harlan Sprague-Dawley, Inc. (Madison, WI). To generate hybrid F1 progeny, F344 females were mated with Cop males. Female (F344 X Cop) F1 progeny were then crossed with F344 males to generate the N2 backcross progeny. All animals were maintained on a standard diet of rat chow and acidified water *ad libitum* and housed in an approved facility with climate control and a 12-h light-dark cycle. A relational database developed in our laboratory was used to track all breeding, treatments, tumor sites, incidence, latencies, phenotypes and genotypes.

### Carcinogen Treatment and Phenotyping

Female N2 and F344 rats were injected with a single intraperitoneal dose of NMU (50 mg/kg body weight) at 50–55 days of age. NMU (Ash-Stevens, Detroit, MI) was prepared as a stock solution of 10 mg/ml in a 0.9% NaCl solution acidified to pH 5.0 with acetic acid. Fresh solutions of NMU were prepared every 30 minutes. Following carcinogen exposure, N2 progeny were maintained *ad libitum* on acidified water and a high fat diet (rat chow supplemented with 30% fat) to promote tumor growth. Animals were palpated for tumor formation on a weekly basis, and animals were euthanized when moribund or when tumors reached a diameter of one centimeter. Mammary tumors and control normal mammary tissue from tumor-bearing animals were collected, frozen in liquid nitrogen and stored at –80°C. A section of each tumor was fixed in formalin and embedded in paraffin. Sections of each tumor specimen were stained with hematoxylin and eosin for histopathological analysis. The numbers and latency of mammary carcinomas arising were recorded for each animal (Figure S1 in File S1).

### Isolation of Genomic DNA and Genotyping

Genomic DNA was isolated from mammary tissues using the Qiagen DNeasy 96 Tissue Kit (QIAGEN Inc. Valencia, CA). Informative Simple Tandem Repeat (STR) markers that have different lengths in the Cop and F344 strains were selected from the Rat Genome Database (http://rgd.mcw.edu/). Swept radii were calculated using the Haldane mapping functions, and used to estimate the optimum number of markers and N2 backcross progeny required for complete coverage of all 20 autosomes genome at the 95% confidence limits. No markers on the rat X chromosomes were included in the analysis. For low-resolution mapping, 77 polymorphic markers (Table S1 in File S1) across the genome were genotyped in 99 female N2 backcross progeny that were also phenotyped for susceptibility to NMU-induced mammary carcinogenesis. The longest distances in centiMorgan (cM) between markers and the farthest distance from the end of the map are listed in Table S2 in File S1. For high-resolution mapping (1–2 cM intervals whenever possible), we used informative markers from in and around the intervals that yielded significant or suggestive linkage scores in the low-resolution mapping.

PCR primers for STR markers were purchased from Research Genetics, Inc. (Madison, WI). The PCR-based genotyping assays were performed using one of two methods. If the lengths of the STRs differed by eight or more base pairs between the F344 and Cop alleles, the PCR reactions were performed with the unlabeled primers purchased from Research Genetics, and the genotypes were determined by amplicon length using polyacrylamide gel electrophoresis (PAGE). For STRs differing by six or less base pairs, PCR reactions were performed with the fluorescently labeled primers, and the genotypes were determined on an ABI PRISM® 3100 Genetic Analyzer System equipped with a G5 filter. The fluorescence data were analyzed with Genescan software, and the genotype calls were determined with Genotyper software (ABI). All the allelic calls, including those from regular PAGE analysis, were independently verified by at least one other person before importing into the database for further analysis.

### Genetic Linkage Statistical Analyses

Linkage analyses were performed with MapManager QTX [16] using tumor number as the quantitative trait. Genetic interactions and associations were tested using two-way ANOVA, χ^2^ tests and logistic analysis models using the SAS v.8.0 statistical software package (SAS Institute, Inc).

### Fluorescence *In Situ* Hybridization (FISH)

Fibroblasts from the BN/SsNHsdMcw, F344, Cop and F1 rat strains were cultured, blocked in mitosis with colcemid, and harvested to prepare metaphase spreads according to published procedures. Rat BAC clones that included the target STR marker sequences were purchased from the Children’s Hospital Oakland Research Institute (CHORI-230 BAC library) and verified by PCR amplification of the STR sequence. BAC DNA was isolated on an Autogen 740 system, biotinylated by nick-translation, hybridized to metaphase spreads in the presence of excess unlabeled rat Cot1 DNA, and detected with avidin-FITC as described elsewhere [17]. The chromosomes were QFH-banded by DAPI staining. DAPI and FITC images were collected separately, but in registration. Hybridization signals were analyzed in at least five, and more typically 10, metaphase cells per probe.

### PCR Amplification and Sequencing of Candidate *Mcs* Genes

PCR primers for amplification of selected candidate genes are shown in Table S3 in File S1 for *Brca2* gene and Table S4 in File S1 for the rat *Fry* gene. For the longer exons, primers were designed to generate two or more overlapping amplicons. Following RNA extraction from normal mammary glands of the F344 and COP rats using a Qiagen RNeasy Maxi Kit (Qiagen Inc. Valencia, CA), cDNA was produced using reverse transcription reaction according to SuperScript™ II Reverse Transcriptase instructions (Invitrogen, Carlsbad, CA). The amplified PCR products were separated by agarose gel electrophoresis (0.8–1.5%), and PCR products excised from the gel and purified with Qiagen gel extraction kits (Qiagen Inc. Valencia, CA).

DNA sequencing was performed using the Big Dye Cycle Sequencing Protocol (PerkinElmer Biosystems Inc. Boston, MA). Each PCR product was sequenced on both strands with the same primers used in the PCR amplification reaction. For the longer PCR products, additional sequencing primers were designed to generate overlapping amplicons. The labeled sequencing reaction products were separated on an automated, fluorescence-based sequencer (Model 377 from Applied Biosystems, Foster City, CA). Automated base calls were reviewed by visual inspection. Sequences were assembled using Sequencher 4.2 software (Gene Codes Corporation, Ann Arbor, MI).

### Northern Blot Analysis

RNA was extracted from the normal mammary glands of F344 and COP rat strains using a Qiagen RNeasy maxi kit (Qiagen Inc. Valencia, CA). Total RNA samples (30 µg) were separated by electrophoresis on a 0.8% (w/v) formaldehyde/agarose gel, transferred to Hybond-N nylon membranes (Amersham Pharmacia Biotech) and probed with a [α-32P]-dCTP labeled Brca2 probe. Following hybridization and washing, autoradiography was performed by exposure to X-ray film.

### Cell Culture

Cell cultures were maintained at 37°C in a humidified atmosphere of 5% CO_2_ and 95% air. MCF-10A cells were cultured in Mammary Epithelium Basal Medium (MEBM), supplemented with gentomicin sulfate, amphotericin-B, 20 ng/ml human epidermal growth factor, 10 µg/ml insulin, 5 µg/ml hydrocortisone and bovine pituitary extract using MEGM SingleQuots (Lonza, Allendale, NJ). HCC1954, T47D, MCF7 and MDA-MB-231 cells were cultured in Advanced DMEM with 10% heat inactivated FBS with 100 µg/ml penicillin-streptomycin solution.

### Semiquantitative Reverse Transcription (RT)-PCR

Tissues from normal Cop and F344 rats as well as mammary tumors and control mammary tissue from NMU-treated F344 rat were collected. Total RNA was isolated from these tissues and human breast cancer cell lines using the Qiagen RNAEasy Mini kit (Qiagen, Valencia, CA). Reverse transcription reactions were performed with the SuperScript™ II Reverse Transcriptase kit (Invitrogen, Carlsbad, CA). The PCR conditions for DNA amplification in the linear range were established on a GeneAmp PCR System 7600 (PerkinElmer, Wellesley, MA). Platinum® Taq DNA Polymerase (Invitrogen, Carlsbad, CA) and primers for the Rat *Fry* gene (5- GGCCTGTTCATAGACTCT -3 and 5- TCCAAGAACAACGCTCCA -3), rat *β-actin* (5-AGTACCCCATTGAACACG -3 and 5-GAGGTCTTTACGGATGTC -3), human *FRY* gene (5-GGTTACAGGAGCCGTTCT -3 and 5- CATCGGCCTGTCGTCATC-3) and human *β*-actin (5-CATGAAGATCCTCACCGA -3 and 5- AAGGGACTTCCTGTAACA-3) were used for DNA amplification. RT-PCR products were analyzed on 1% agarose gels, and expression was quantified using Image J software (NIH, Bethesda, MD) and normalized to β-actin.

### Quantitative Real-time PCR Analysis

Total RNA was extracted from three independent cultures exponentially growing cells (70% confluence) using Qiagen RNeasy Mini protocol. Relative expression levels were meaured using qunatitative Real-time PCR chain reactions (QRT-PCR) and SYBR® Green intercalation to quantify amplification. Sequences of the human FRY specific forward and reverse primers were primers 5′ TCC CAT GTC TGG ATA TTT GC –3′ and 5′ – ATA AGG CCA GGC ATA GCT GA –3′, respectively. All reactions were performed in triplicate (technical replicates) and products verified by gel electrophoresis. CT values were normalized to those of β-actin. Expression levels are shown relative to MCF10A cells. Error bars indicate standard deviations.

### Western Blot Analysis and Validation of the FRY Anti-peptide Antibody

To generate polyclonal antisera against the FRY protein, rabbits were immunized with a FRY-specific peptide conjugated to keyhole lympet hemocyanin. The immunogenic peptide used was chosen from a region of the FRY gene whose predicted amino acid sequence was conserved in mice, rats and humans.

Total cellular extracts (30 µg protein) were separated by electrophoresis in pre-cast 7.5% Tris-HCl gels (Bio-Rad, Hercules, CA) and transferred to Immobilon–FL PVDF membranes (Millipore, Billerica, MA). The membranes were incubated with this validated anti-FRY antiserum (1∶10,000 dilution) overnight at 4°C. The blots were then rinsed three times with TTBS for 10 min, 7 min, and 5 min, respectively, rinsed briefly with TBS and incubated with LI-COR anti-rabbit secondary antibody for 45 minutes under gentle agitation at 4°C. Blots were developed using the LI-COR Odyssey Infrared Imaging System. The FRY protein was verified through blocking the antibody (or the pre-serum) with the peptide. The antiserum detected a 280 kDa protein in rat kidney (not shown), while the molecular weight was ∼120 kDa in rat and human mammary cells, suggesting tissue-specific posttranscriptional regulation. FRY protein levels were normalized to β-actin.

### 5′ and 3′ RACE Reaction

Mammary tissues from normal Cop and F344 rats were collected. Total RNA was isolated from these tissues using the Qiagen RNAEasy Mini kit (Qiagen, Valencia, CA). The 3′ and 5′ RACE were used to determine the open reading frame (ORF) of the rat *Fry* mRNA sequence. Amplifications were carried out with the Marathon cDNA Amplification Kit (BD-Clontech, Palo Alto, CA). Two gene-specific primers, 5′ - GTGGGTCAACGTCGTCTGCCGCTAC - 3′ (3′-RACE) and 5′ - TCAAATAGTCTCGCTGCTGCTCGTCAC - 3 ′ (5′-RACE), were designed from the nucleotide sequences within the sequenced DNA fragments. Double stranded cDNA prepared using the Marathon cDNA Amplification Kit was amplified and cloned using the TOPO TA kit for DNA sequencing.

### SNAP Analysis

The SNAP (Screening for Non-Acceptable Polymorphisms) computational tool predicts the functional consequences of single amino acid substitutions in both binary (neutral/non-neutral, with respect to wild type function) and scored from −100 to +100, where negative predictions are neutral, positive are non-neutral, and higher absolute values of scores indicate higher reliability of the binary prediction [18]. Using SNAP, we determined whether the non-synonymous SNPs in the Fisher 344 rat strain are indicative of functional effects when present in the rat as well as the human *FRY* genes. The SNAP output scores were evaluated for each amino acid substitution at the codons in question. Additionally, we assessed the likely functional (as opposed to structural) importance of each amino acid in the protein sequence by computing the SNAP-SubMat score – for each wild-type residue, the *average* of all SNAP scores of substitutions allowed by the BLOSUM62-matrix at cutoff >0 [19]. SNAP prediction of non-neutral functional effects for substitutions expected to be functionally neutral (as indicated by common, BLOSUM62-defined exchangeability of the involved residues in evolution) is indicative of the functional importance of the protein sequence position in question. As with regular SNAP scores, SNAP-SubMat scores <0 indicate that a specific sequence position is not likely functionally significant, and a score >0 indicates that this location is probably functionally significant.

## Results

### Genome-wide Screen for *Mcs* (QTL) Loci

**Figure 1:**
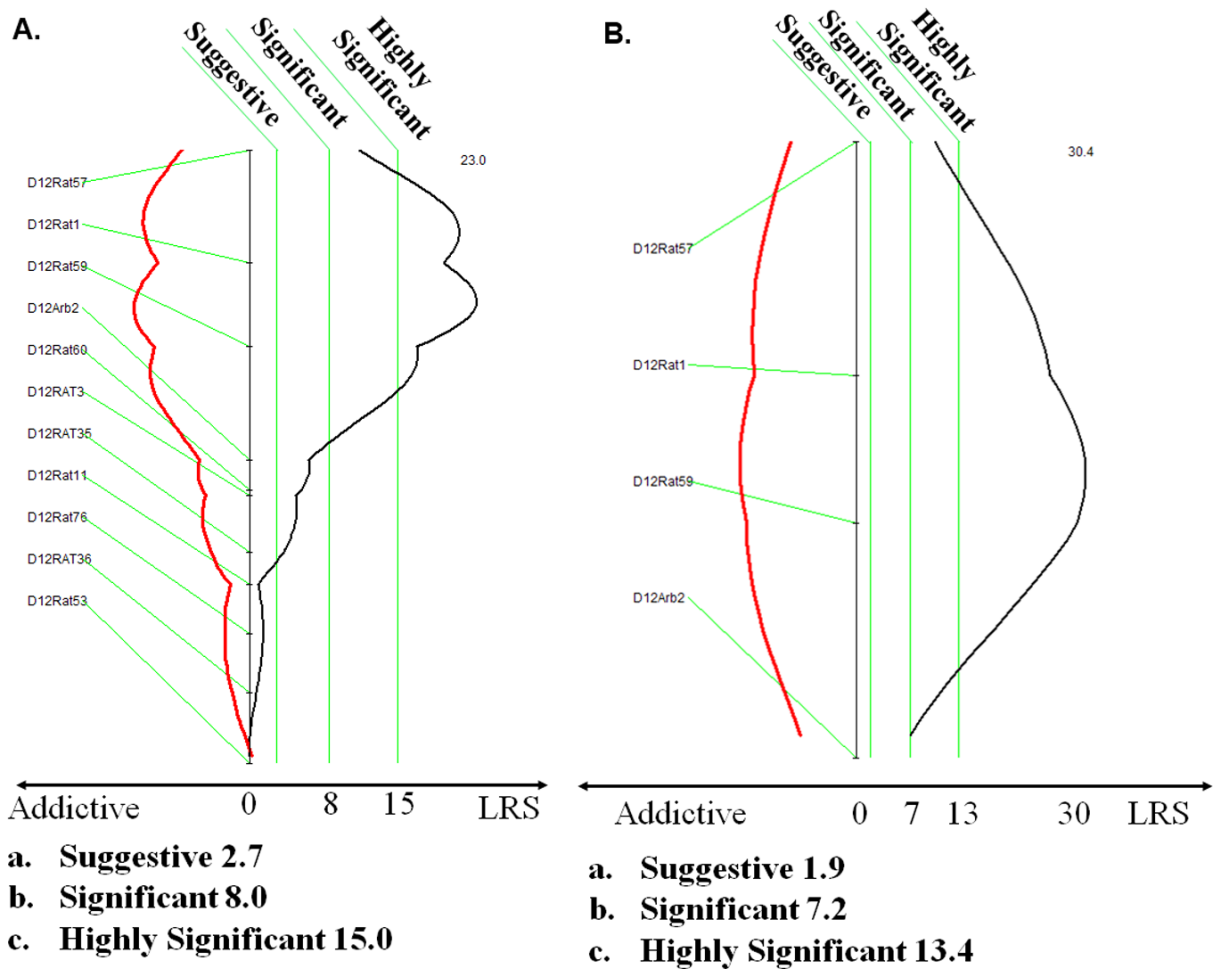
Interval mapping of putative quantitative trait loci (QTL) on rat chromosomes 12 that confer susceptibility to NMU-induced mammary carcinogenesis. The genetic map positions of polymorphic STR markers are indicated by hatch marks and are designated to the left. Genetic distances between markers in centiMorgans are drawn to scale within each panel. The calculated Likelihood Ration Statistic (LRS) values for linkage of the Quantitative trait (measured as log of tumor numbers) within chromosomal intervals are plotted as X to the right (black line). **A)** Linkage calculated on chromosome 12, using 99 animals genotyped at the markers indicated on the left. **B)** Linkage on chromosome 12, as calculated using 324 animals genotyped at the markers indicated on the left. The vertical green lines indicate the suggestive (LRS = 2.7 for Panels A; LRS = 1.9 for Panel B), significant (LRS = 8.0 for Panels A; LRS = 7.2 for Panels B), and highly significant (LRS = 15.0 for Panels A; LRS = 13.4 for Panels B) thresholds as calculated by permutation studies. The additive effect functions are represented as dotted red lines, with negative values to the left and positive values to the right.

We performed linkage analysis using a genetic backcross between two rat strains with differential susceptibility to NMU-induced mammary carcinomas (the resistant Cop and the susceptible F344 strains). We selected a panel of 77 informative STR markers (Table S1 in File S1) that provided an estimated 95% coverage of the twenty rat autosomes (Table S2 in File S1). Low-resolution linkage of 50 N2 progeny with and 49 without mammary tumors provided suggestive evidence for linkage of tumor susceptibility to the short arm of rat chromosome 12 (RNO12) (normal font in Table 1). About 58% of the N2 progeny developed mammary tumors over the course of one year after NMU exposure (Figure S1 in File S1). We used the number of mammary tumors induced within the first 210 days after NMU exposure as the quantitative trait (Figure S1 in File S1). We initiated high-resolution QTL mapping using an additional 225 female N2 progeny. All 324 of the NMU-treated N2 progeny were genotyped at all informative markers (bold font in Table 1) from the candidate locus on RNO12. Markers bounded by and including D12Rat57 and D12Arb2 yielded highly significant linkage, with a maximum LOD score of 8.6 at the D12Rat59 marker (Table 1), demonstrated the presence of a new *Mcs* locus. Interval mapping [20] also indicated highly significant linkage to the same intervals on RNO12 (Figure 1 A and B) confirming the presence of a new *Mcs* locus. The direction of the additive effect confirmed that the presence of the Cop *Fry* allele of the *Mcs* was negatively correlated with susceptibility. This new susceptibility locus on RNO12 was designated as *Mcs30* (QTL30) and was assigned the RGD ID: 7243862.

**Table 1:** Calculated LOD scores for markers on rat chromosome 12.

Marker Name	Number of Rats Genotyped	LOD
**D12Rat57**	**324**	**3.3**
**D12Rat1**	**324**	**6**
**D12Rat59**	**324**	**8.6**
**D12Arb2**	**324**	**2.1**
D12Rat3	99	2.7
D12Rat35	99	0.8
D12Rat36	99	0.5

Note: LOD, logarithm of the odds ratio.

### FISH Mapping of the Putative *Mcs* Locus on RNO12

At the time we performed these studies, both genetic linkage [21], [22] and the rat radiation hybrid mapping [23] placed all the STRs that defined the *Mcs30* locus on the short arm of RNO12 [24]. The D12Rat59 marker was also depicted within the short arm of RNO12 on the UCSC Genome browser (http://genome.ucsc.edu/). However, rat chromosome 12p is a satellite, comprised of tandem repeat sequences that show strain-specific variations in length [25], [26]. Using several Bacterial Artificial Chromosome (BAC) clones harboring the D12Rat59 marker (LOD ∼8.6), we physically mapped the *Mcs30* locus by fluorescence *in situ* hybridization (FISH). We used the sequence of the highly linked D12Rat59 marker (LOD ∼8.6) for a BLAST search of the available rat genome sequences, and assembled a contig comprising three overlapping BACs: CH230-85G15; CH230-381M14; and CH230-275K13 (Figure S2 in File S1).

**Figure 2:**
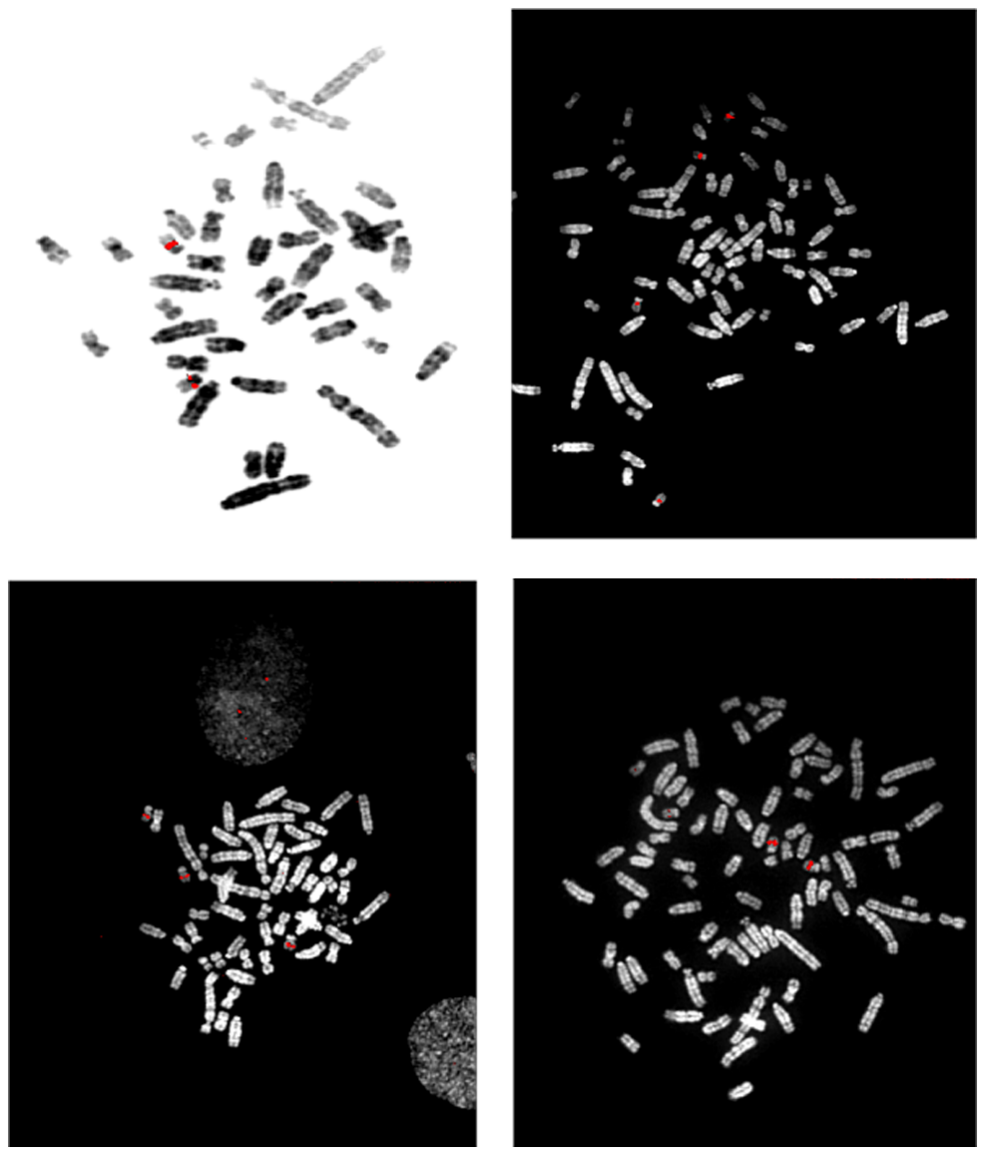
FISH mapping of BAC CH230-85G15, containing D12RAT59, to rat chromosome 12q11-q12. Metaphase chromosome spreads were prepared from skin fibroblasts isolated from the BN/SsNHsdMcw rat strain and from cultured embryo fibroblast isolated from (F433 X Cop) F1 progeny rats. Metaphase chromosomes were hybridized with fluorescently-labeled BAC CH230-85G15, which contains D12RAT59, the marker on RNO12 showing tightest linkage to *Mcs30*. The hybridization signals are pseudo-colored red for clarity, and DAPI banding is displayed as either grey or white values. Other BACs containing D12Rat59 were also hybridized to 12q11-12 (See Table 2 and Figure S3 in File S1).

Each of these BAC clones was physically mapped by fluorescence *in situ* hybridization (FISH) to metaphase chromosomes prepared from embryo fibroblasts of Cop, F344, and (F344 X Cop)F1 hybrid strains, as well as the BN/SsNHsdMcw strain used for rat genomic sequencing (Figure S3 in File S1) [24]. BACs CH230-123F8 and 151L24 were used as controls, which include the *EPO* and *PAI1A1* gene sequences, respectively to RNO12q12 and RNO12q11 (Table 2) [27], [28]. All of the BACs tested were FISH-mapped to the long arm of rat chromosome 12. BAC CH230-85G15, which includes the highly linked D12Rat59 marker, mapped to the 12q11-12 region of RNO12, close to the centromere (Figure 2). FISH analyses performed using embryo fibroblasts from the F344 and Cop strains, as well as F1 hybrids yielded identical results. Together these results indicated that the *Mcs30* locus mapped to the centromeric region on the long arm of rat chromosome 12 (RNO12q). FISH analysis did not reveal any gross differences between the Cop and F344 in the region defined by the BACs, although there is slight heteromorphism in the pericentromeric region proximal to the FISH signals.

**Table 2:** BACs used for FISH. BACS containing sequences from the D12Rat1, D12Rat59, Epo and PAI1A1were used from physical mapping of loci to Rat chromosome 12.

BAC Identification	Sequences withinBAC	Chromosomal Localization
CH230-152N10	D12Rat1	RNO12q11-12
CH230-85G15	D12 Rat59	RNO12q11-12
CH230-381M14	D12Rat59	RNO12q11-12
CH230-275K13	D12 Rat59	RNO12q11-12
CH230-123F8	PAI1A1	RNO12q12
CH230-151L24	EPO	RNO12q12

### The *Mcs* Gene on Rat Chromosome 12 is Distinct from *Brca2*

**Figure 3:**
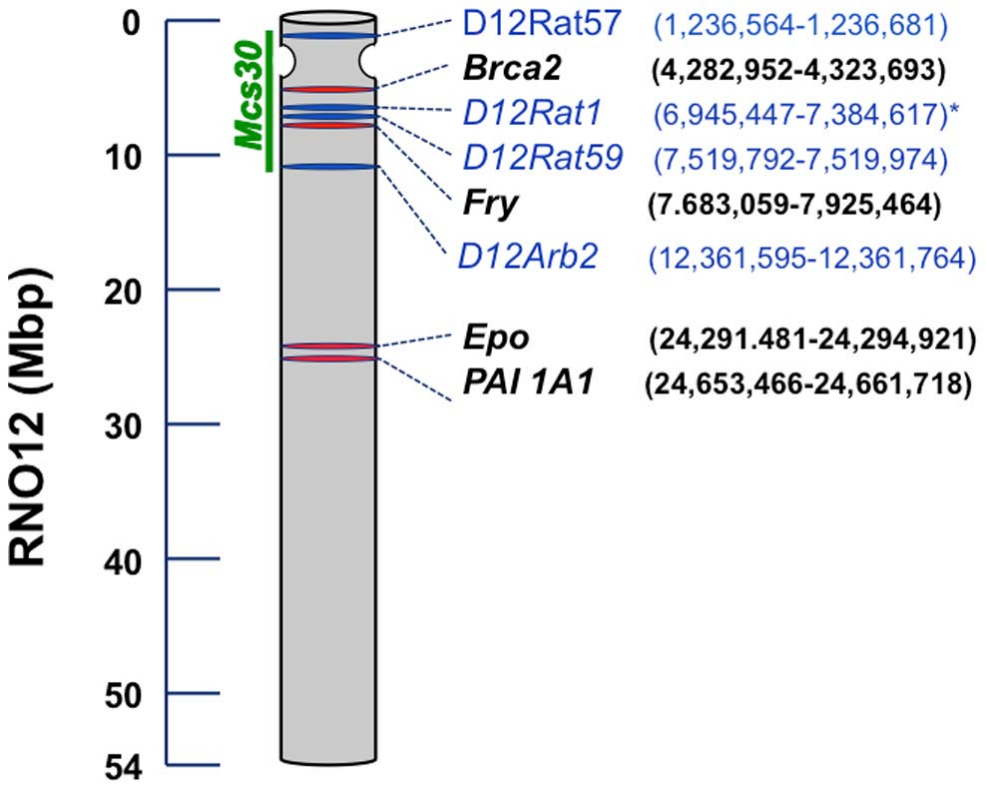
Ideogram of rat Chromosome 12 (RNO12). Ideogram shows the relative locations (drawn to scale) and nucleotide positions (in brackets) for STR markers (blue bars and font) and genes (red bars and bold black font). The placement of genes and markers is based on the draft rat genome sequence (RGD Build 5.1 Assembly (Annotation Release103)) The green bar to the left indicates the position of the 11 Mbp *Mcs30* locus (QTL30). The position of the centromere is proposed on the basis of FISH mapping, which places D12 Rat1 on RNO12p11. * Although D12Rat1 is also present on RNO1 (RGD Build 5.1 Assembly (Annotation Release103)), the sequence on RON1 is later significantly shorter (93bp) than the sequence on RNO12 (137 bp). Variants of the latter were used for mapping.

The *Mcs30* locus comprises approximately 12 Mbp on the long arm of RNO12 that probably incudes the centromere (Figure 3). A comparison of the draft human and rat genome sequences indicated that the *Mcs30* locus includes a 5.6 cM region on RNO12q whose synteny is conserved on human chromosome 13q12 to 13q13. Mcs30 harbors at least 186 genes (RGD Rat Genome V3.4 Assembly, down loaded on 5/19/13). We used available functional data (e.g., data from KEGG, GeneCards, etc.) to select several candidate genes whose functions could be related to cancer susceptibility. These candidates included the rat orthologs of the human *BRCA2* gene, the *Stard13* gene, also known as *DLC2* (Deleted in Liver Cancer 2), and the insulin receptor (*Insr*).

*Brca2* gene is ∼0.6 Mbp distal to the highly linked D12rat59 marker, raising the possibility that *Brca2* gene was a *Mcs* gene in QTL30. A previous study demonstrated that the known haplotypes within exon 11 of the rat *Brca2* gene did not segregate with the susceptibility phenotype [29]. However, these observations could not rule out the possibility that the rat *Brca2* gene includes haplotype blocks that segregate with susceptibility.

**Figure 4:**
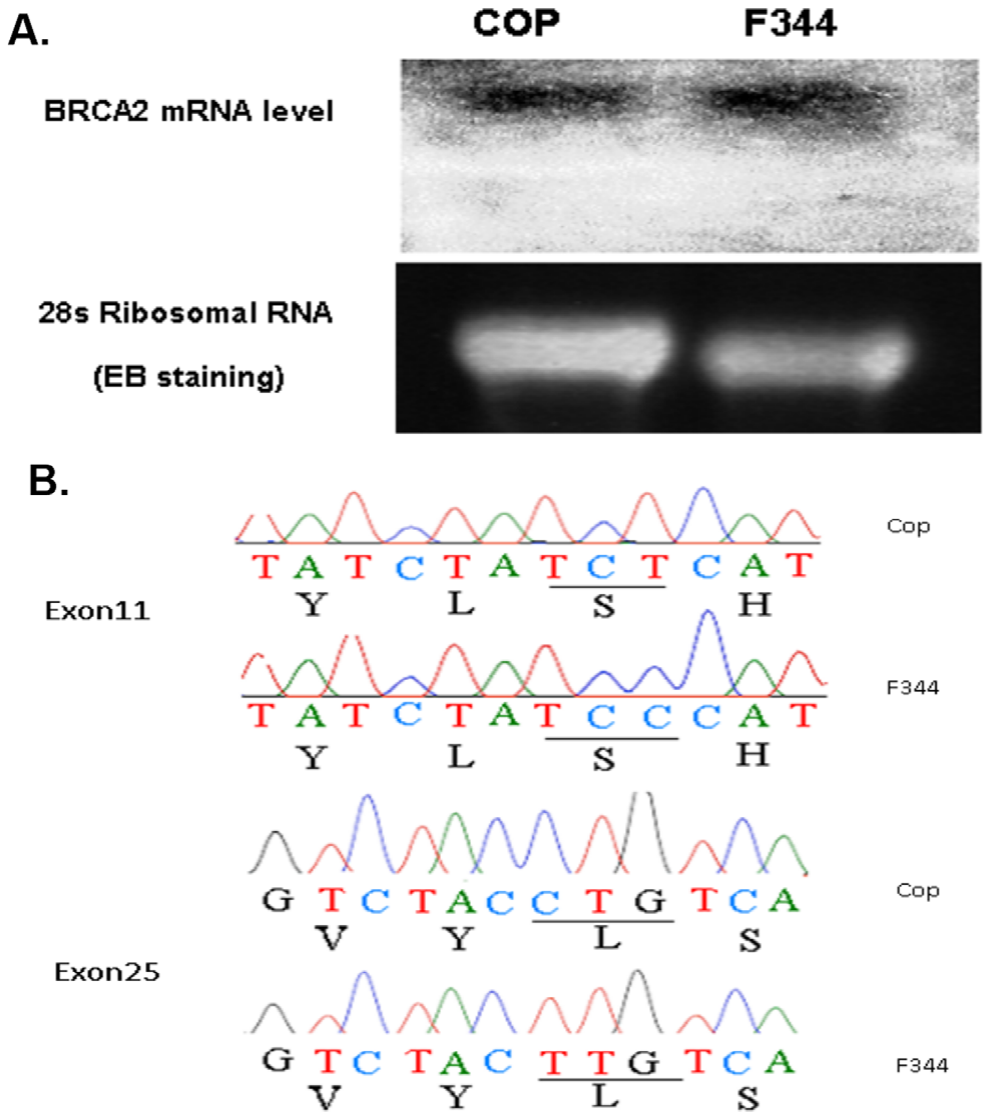
Comparison of *Brca2* mRNA levels in mammary tissue and DNA sequences in Cop and F344 rats. (A) Steady-state levels of the 13 kb Brca2 mRNA were compared by Northern blot analysis. *Brca2* mRNA was expressed at comparable levels in both strains. (B) Comparison of *Brca2* cDNA sequences in Cop and F344 rat strains. DNA sequence analysis detected two synonymous single nucleotide polymorphisms (SNP); a T to C transition in Exon11 and C to T transition in Exon25.

To rule out strain-specific epigenetic silencing, we compared levels of *Brca2* expression in mammary tissue from F344 and Cop rats. Northern blot analysis showed that *Brca2* was expressed at comparable levels in mammary glands of both F344 and Cop rats (Figure 4A). We next asked if the *Brca2* gene from the two strains differed at the nucleotide sequence level (See Table S5 in File S1 for all Accession Numbers). The genomic sequencing results showed that the coding sequence of the *Brca2* genes from the Cop and F344 strains (Genbank accession numbers for *Brca2*: AH014113.1 (Cop) and AH014114.1 (F344)) differed by only two synonymous SNPs at nucleotides 5380 and 9239 (Figure 4B), one of which (nucleotide 5380) was reported in previous study, which also showed that this SNP was not linked to genetic susceptibility [29]. We found no strain-specific sequence differences within the *Brca2* splice junctions or adjacent sequences. Together, these results suggested that differences in the sequence, transcription or splicing rat *Brca2* did not contribute to the susceptibility of the F344 rat strain to NMU-induced mammary carcinogenesis.

We also found no strain-specific alterations in DNA sequence for several other candidate genes within the Mcs locus, including the domain containing *STARD* 13 (*Stard13*) and the insulin receptor (Insr): the sequences of the *Stard13* and *Insr* genes in both the Cop and F344 strains were identical to those previously reported (NM_001109060 and NM_017071.2, respectively) (See Table S5 in File S1 for all Accession Numbers). Moreover, the mRNA levels of both genes were comparable between Cop and F344 rat, and NMU-treatment did not affect the expression levels of *Insr* and *Stard13* (Figure S4 in File S1).

### Analysis of mRNA Sequence and Tissue Specific Expression of the Rat *Fry* Gene

The *Mcs30* locus also included the *Fry* gene, previously designated as a hypothetical gene similar to the human CG003 (predicted) gene. Since the rat *Fry* ortholog was only 30 Kbp distal to the D12Rat 59 STR marker, we decided to evaluate its potential role in genetic susceptibility. We designed a series of PCR primers to amplify and sequence overlapping fragments comprising the predicted open reading frame of the rat ortholog (Table S4 in File S1). Sequencing failed to reveal a transcription start site within the predicted open reading frame. We therefore preformed 5′ and 3′ RACE (rapid amplification of cDNA ends) using mRNA extracted from mammary tissue. The results demonstrated that the transcript in rat mammary tissue includes 10792 nucleotides encoding *Fry*, the rat ortholog of the *Drosophila furry* gene. Rat *Fry* mRNA encodes a predicted protein comprising 3011 amino acids.

**Figure 5:**
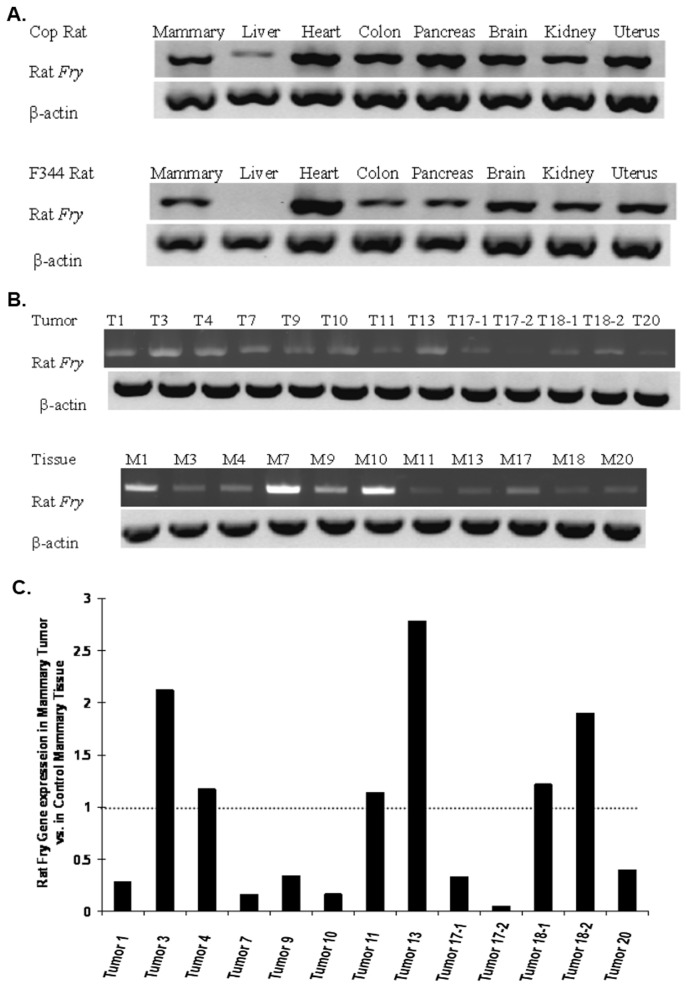
Expression of rat *Fry* mRNA in rat tissues and mammary tumors. Semi-quantitative RT-PCR analysis of steady-state *Fry* mRNA levels in somatic tissues of Cop and F344 rats. β-actin expression was used for normalization (lower panel). (**B**) Semi-quantitative RT-PCR analysis of steady-state Fry mRNA levels in normal mammary tissue, mammary tumors and normal mammary tissue collected from the matched tumor bearing F344 rats. β-actin mRNA of rat was used for normalization (lower panel). (C) Image densitometry in (B) was quantified using Image J software.

RT-PCR analyses demonstrated that the rat *Fry* gene is highly expressed in the heart and brain, with relatively lower expression levels in several other tissues including the mammary gland, kidney, pancreas, colon, uterus, and liver (Figure 5 A). We found no-strain specific differences in the expression of the *Fry* gene in any of the organs tested from Cop and F344 rat, including mammary glands.

We next compared *Fry* expression levels in normal rat mammary tissue with those in mammary carcinomas collected from same rat. The results showed that the rat *Fry* gene was expressed at low levels in a majority of mammary tumors relative to mammary tissue of age-matched control rats (Figure 5B and 5C).

### The Genome of the Inbred F344 Rat is Homozygous for the Mutant *Fry* Allele

Since there were no strain-specific differences in the expression of *Fry* in mammary cells, we asked if the F344 *Fry* gene harbors any loss of function mutations. We sequenced the *Fry* gene both in the Cop (NCBI Accession EU563851) and F344 (NCBI Accession EU563850) rat strains. DNA sequence comparison of the *Fry* gene identified 22 SNPs, two of which were non-synonymous. The first of these (codon 661) substitutes an Aspartic acid (D) residue in the Cop protein with a Glutamic acid (E) in F344 protein. The SNP in codon 2170 replaces an Alanine (A) in the Cop protein with a Serine (S) residue in the F344 protein.

**Figure 6:**
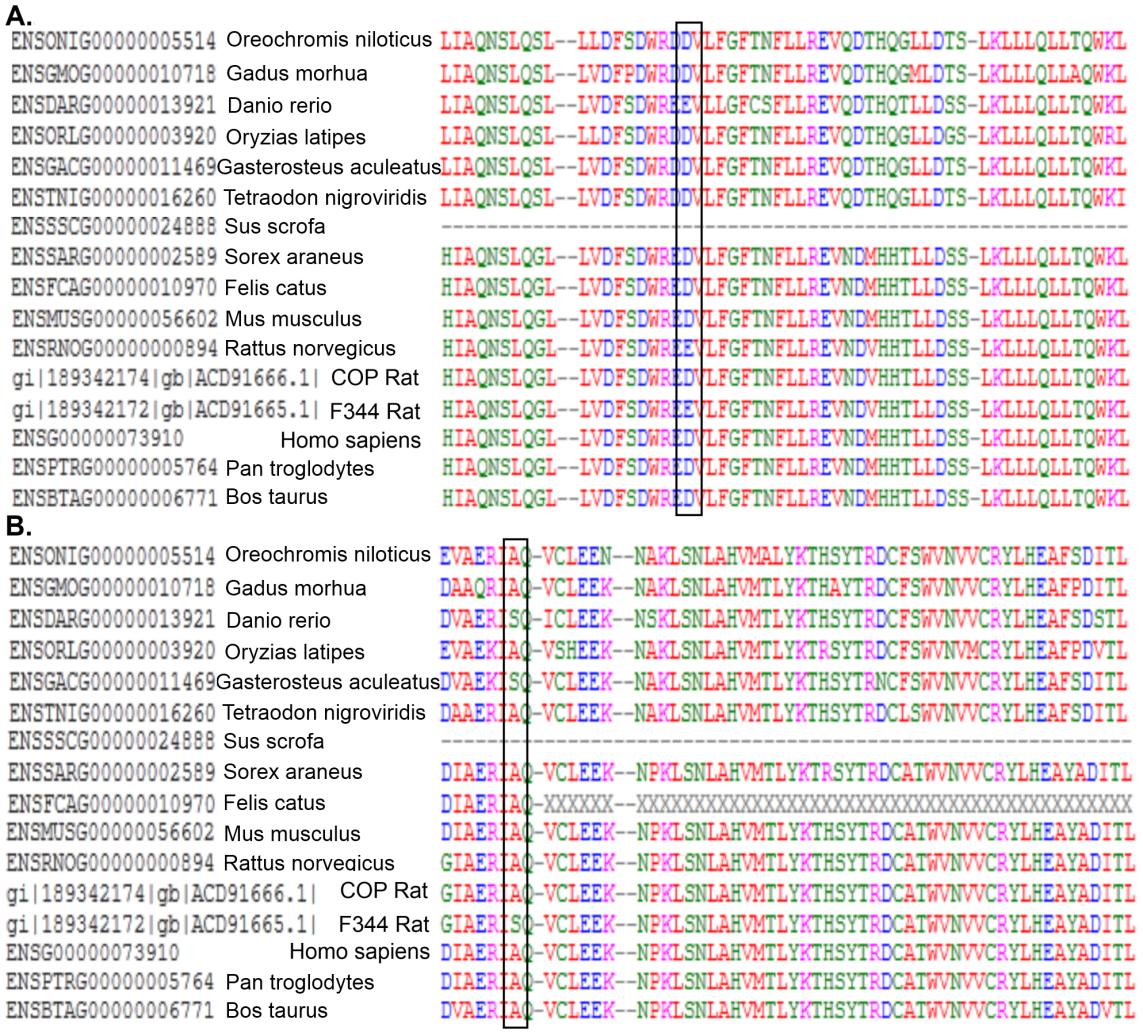
Alignment of Proteins Encoded by *FRY* gene orthologs from different species. The *Fry* allele present in the F344 allele carries two non-synonymous SNPS at amino acid residues, codon 661(Panel A) and 2170 (Panel B), are conserved in a majority of FRY orthologs (showing part of aligned results due to space limit). Strain specific SNPs present in the Fischer 344 rat strain replaces Aspartic Acid (D) and Alanine(A) residues present in Cop rat with amino acid Glutamic Acid (E) and Serine(S) in F344 rat, at amino acid 661 and 2170, respectively.

To investigate if these amino acid substitutions occurred in conserved sequences, we (multiple sequence) aligned the sequences of all vertebrate orthologs of the fry gene (OrthoDB, group id EOG6PCBZN) [30] and the COP and F344 strain sequences using ClustalOmega [31]. The results showed that these two amino acid residues were identical to those of the resistant Cop strains in most species tested (Figure 6), which harbors conservative amino acid substitutions, and a few orthologs are missing this region altogether. These observations are consistent with the possibility that the variant allele present in F344 rat alters the function of the Fry protein. Nonetheless, if the alignment is performed with protein families rather than the FRY sequence, this region of the FRY protein was not explicitly conserved.

We therefore used SNAP [18] to further investigate whether these non-synonymous SNPs in the Fisher 344 rat strain are indicative of functional effects, or if they were to occur in the human protein. The human FRY protein sequence was taken from the UniProt [32] (Q5TBA9; FRY_HUMAN). The SNAP scores for an amino acid change at rat Fry codon 661 (where the F344 rat had an Aspartic acid to Glutamic acid mutation) in the rat and human protein were −51 and −44, respectively, indicating that this mutation is not likely to cause functional consequences. SNAP-SubMat scores of 2 and −11 for position 661 in the rat and human proteins, respectively, further suggested that this amino acid residue may not be functionally significant. We also analyzed the SNAP and SNAP-SubMat scores for the human protein residue 2171, which coincided with the F344 rat Alanine to Serine mutation in codon 2170. The SNAP scores for an amino acid substitutions at rat Fry codon 2170 and human FRY codon 2171 were score −74 and −67 respectively, indicating that an amino acid change at this location is also not likely to have functional consequences. The SNAP-SubMat scores of −16 and −37 for amino acid substitutions in this residue in the rat and human proteins, respectively, also indicated that this location is may not be functionally significant in the FRY protein.

SNAP-SubMat scores are a good way to measure functional significance of protein residues within a sequence. Our results indicate that neither of the mutations affects a clearly functional site in the protein. However, SNAP does not asses the functional effects of newly introduced amino acids, such as acquiring *de novo* phosphrylation sites. Since *Drosophila melanogaster* and *Saccharomyces cerevisiae Fry* orthologs regulate the function of serine/threonine protein kinases [15], [33], we next asked if the introduction of Serine residue created a *de novo* phosphorylation site in the mutant Fry protein in F344 rats. Using the NetPhos2.0 [34] phosphorylation site prediction software, we confirmed that the serine residue in the F344 rat *Fry* sequence was within a peptide sequence with a 98.6% probability of being a substrate for several protein kinases (Table 3). Moreover, replacing the alanine in the human protein with a serine, also produced a predicted phosphorylation site.

**Table 3:** Prediction of a novel phosphorylation site in the F344 Fry allele using NetPhos2.0 phosphorylation prediction software.

NetPhos2.0 Predict Software				
Name	Position	Context	Score	Predicted
Sequence	2170	AERISQVCL	0.986	*S*
Site	Kinase			Score
**S-2170**	**protein kinase, DNA activated, catalytic polypeptide (predicted) [** ***Rattus norvegicus*** **]**			**0.51**
**S-2170**	**ataxia telangiectasia mutated homolog (human) (mapped) [** ***Rattus norvegicus*** **]**			**0.55**
**S-2170**	**protein kinase, cAMP-dependent, catalytic, [** ***Rattus norvegicus*** **]**			**0.60**

### Altered Expression and Mutation of FRY in Human Breast Cancer Cell Lines

**Figure 7:**
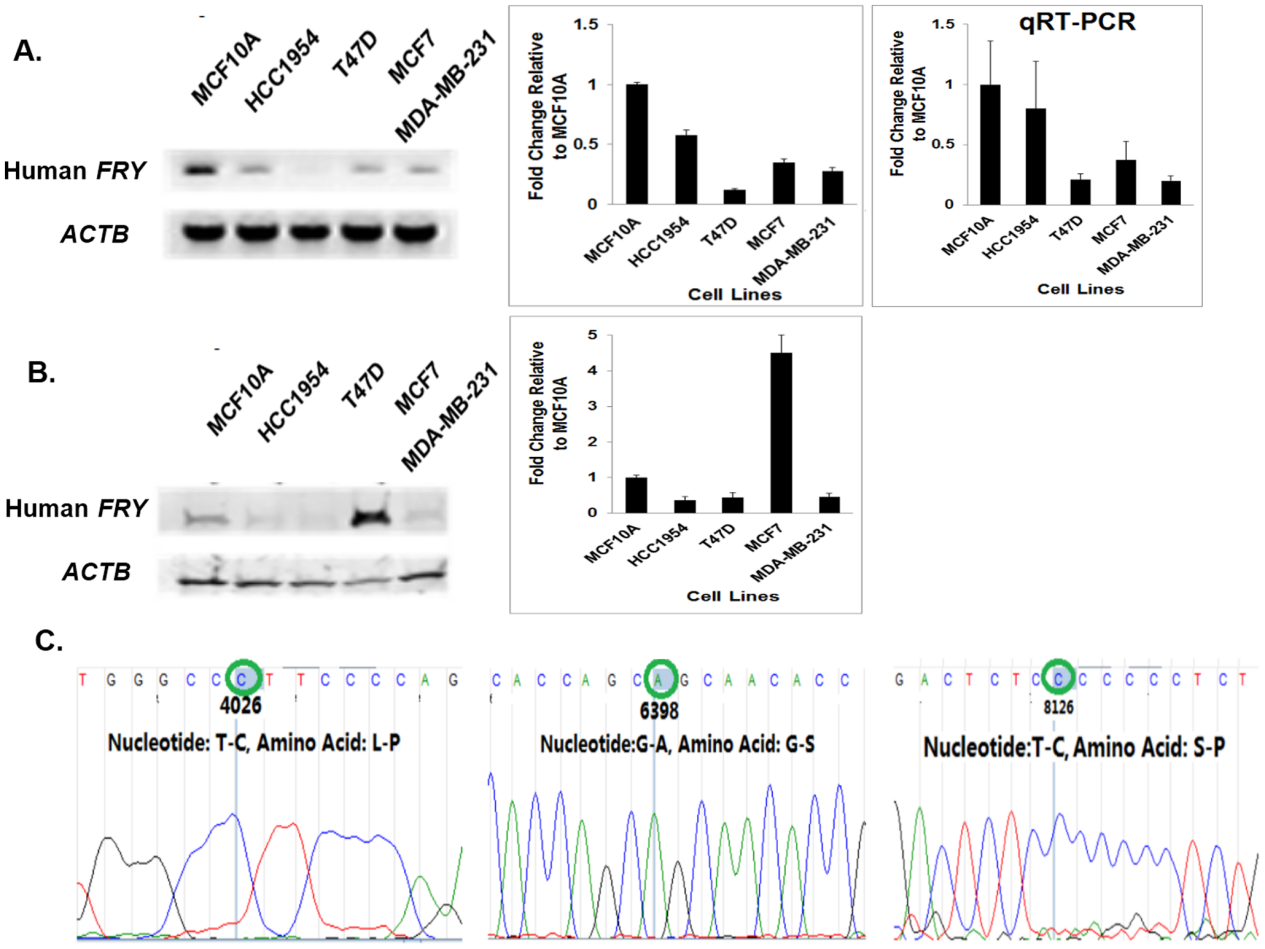
FRY expression and/or function are reduced in breast cancer cell lines relative to the nontransformed MCF-10A human mammary epithelial cell line. Expression of FRY mRNA and protein in the non-tumorigenic, MCF-10A human mammary epithelial cell line was compared to those in ER-negative (HCC1954, MDA-MB-231) and ER-positive (T47D, MCF7) breast cancer cell lines. (**A**) Comparison of *FRY* mRNA expression levels by semiquantitative RT-PCR. Steady-state Fry mRNA levels were reduced in all 4 of 4 breast cancer cell lines relative to non-transformed MCF-10A cells. The results were further confirmed by qRT-PCR. (**B**) Comparison of FRY protein expression levels by Western blot analysis. Steady-state levels of FRY protein were reduced in 3 of 4 breast cancer cell lines which also showed reduced mRNA levels. Only the MCF-7 cell lines showed elevated levels of FRY protein despite having a reduced level of mRNA relative to MCF-10A cells, suggesting altered posttranscriptional regulation. (**C**) MCF-7 breast cancer cells harbor a mutated *FRY* gene. cDNA sequencing was used to compare the sequence of the *FRY* coding sequence in MCF-7 cells to the reference human genome sequence. The analysis identified a total of 10 SNPs, three of which led to the non-synonymous amino acid substitution indicated.

We next measured human *FRY* gene mRNA levels in several human breast cancer cell lines. As shown in Figure 7A, the level of FRY mRNA expression in all breast cancer cell lines evaluated was reduced by at least 40% relative to the non-tumorigenic MCF10A human mammary epithelial cell line. This result was further confirmed by qRT-PCR analysis. FRY protein levels were decreased in 3 of 4 breast cancer lines evaluated (Figure 7B). Although we observed a higher level of FRY protein in the MCF-7 breast cancer cell line, our studies showed that the FRY gene is mutated and probably dysfunctional in this cell line. DNA sequence analysis showed that the coding sequence of the *FRY* gene in the MCF7 cell line harbors 10 SNPs relative to the sequence published in the NCBI database (NM_023037.2), four of which have been previously reported in the NCBI database. Among these 10 SNPS are three non-synonymous changes (Figure 7C). The Leucine (L) to Proline (P) substitution at amino acid 1177 of the MCF7 FRY protein has a SNAP score +18, indicating that the substitution is highly likely associated with a functionally disruption. The SNP in codon 1968 changes the amino acid from Glycine (G) to Serine (S); the SNP in codon 2544 substitutes a serine (S) with a Proline (P) in MCF7 FRY protein (Figure 7C). However, SNAP analysis suggested that these two substitutions may not lead to functional changes. Taken together, our observations in human and rat mammary tumor cells are consistent with the possibility that decreased FRY activity and/or function is associated with an increased risk of carcinogenesis.

## Discussion

Over the past two decades investigators have made significant progress in elucidating the genetic basis for the differential susceptibility of inbred rat strains to mammary carcinogenesis, identifying 29 susceptibility (*Mcs*) loci [6], [7], [8], [11], [12]. In the present study, we used N2 backcross progeny generated by mating female (F344 X Cop) F1 progeny with F344 males to identify additional *Mcs* loci by linkage analysis. All informative markers on chromosome 12 within the region bounded by and including the D12Rat57 and D12Arb2 markers yielded highly significant LOD scores, with a maximum (LOD∼8.6) at the D12Rat59 marker. This interval, deemed *Mcs30* (QTL30), encodes almost 200 genes and includes a ∼5.6 Mbp region on the rat chromosome 12 whose synteny is conserved on human chromosome 13q12 to 13q13. We therefore used available data to identify candidate *Mcs* genes within this region, including the rat orthologs of the human *BRCA2* gene, the *Stard13* gene (also known as *DLC2*) that encodes a Rho GTPase activating protein with growth suppressor function [35], [36], and the Insulin Receptor (*Insr*). However, comparison of DNA sequences and mRNA levels indicated that these genes did not differ between strains and hence were unlikely to encode the *Mcs* gene within the *Mcs30* locus RNO12q. We therefore focused our attention on *Fry*, a highly conserved gene, located less than 30 Kbp from distal to the highly linked D12Rat59 STR marker.

We first sequenced and established the entire open reading frame and splice sites of the *Fry* gene in Cop and F344 rat strains. The rat *Fry* gene shared significant sequence similarity with the Drosophila *furry* gene, and was ∼80% similar to human (*FRY*) and mouse (*Fry*) orthologs. Our analysis further revealed a total of 22 single nucleotide polymorphisms between the *Fry* alleles of Cop and F344 strains, two of which resulted in amino acid substitutions in the susceptible F344 relative to the resistant Cop strain. One approach to the identification of functional SNPs is to use comparative genomics [37], which predicts that amino acids important to the normal function of a protein will be highly conserved through evolution and show very little species variation. By contrast, amino acids that have little effect on function will be less conserved and more likely to harbor non-synonymous SNPS in distantly related species. Our analyses indicated that while the F344 SNPs are conserved in FRY othologs, they were not explicitly conserved in protein families with related sequences. Since *Drosophila melanogaster* and *Saccharomyces cerevisiae Fry* orthologs regulate the function of serine/threonine protein kinases [15], [33], it is plausible that the introduction of Serine residue could lead to altered Fry phosphorylation and regulation. Consistent with this possibility, the NetPhos2.0 phosphorylation site prediction software indicated that Ser at residue 2170 creates a *de novo* phosphorylation site in the mutant Fry protein in F344 rats. Semi-quanatitative PCR analysis further indicated *Fry* gene was expressed at reduced levels in mammary tumors when compared to normal mammary of non-tumor bearing rats. Although the findings were not confirmed by the sensitive quantitative PCR techniques such as 5′ endonuclease based QPCR [38], [39], we did use qRT-PCR to confirm that levels of *FRY* mRNA expression are reduced in all human breast cancer lines examined (Figure 7a), indicating that loss of FRY expression is also associated with human breast cancer. More importantly, database mining revealed that FRY mRNA and protein expression are reduced in a human cancers arising from a wide variety of different tissue types, and that that reduced FRY expression is associated with tumor progression and clinical outcomes (Jessica Graham, Xuefeng Ren and Helmut Zarbl, submitted for publication). Together, these findings indicate that the rat Fry gene encodes a *Mcs* gene within the *Mcs30* (QTL30) locus. However, these findings do not preclude the possibility that the *Mcs30* locus harbors additional susceptibility genes.

Although the mammalian *FRY* genes have not been functionally characterized, orthologs in lower eukaryotes regulate cell differentiation. The *D. melanogaster (furry)* and *S. cerevisiae* (*tao3*) orthologs regulate cell morphogenesis, polarity, cell wall biosynthesis, and proliferation and cytokinesis. Fry proteins mediate these processes by interacting with and maintaining the functions of a class of Serine/threonine protein kinase, such as Tricornered and Warts kinases in the *D. melanogaster* and the Cbk1 kinase in *S. cerevisiae* [15], [33]. Orthologs to these kinases in humans are members of the nuclear Dbf2-related protein-kinase (NDR) family of kinases. NDR kinases are essential components of pathways that control important cellular processes, such as epithelial cell morphology and polarity, mitotic exit, cytokinesis, cell proliferation and apoptosis from yeast to humans [40]. Recent studies suggest that NDR kinases may also have tumor suppressor activity [41], [42], indicating that FRY regulates susceptibility to carcinogenesis by regulating the activity of NDR kinases. Significantly, computational approaches indicate that the Fry protein is likely to include an ATP binding domain, suggesting that it may be regulated by ATP binding or have intrinsic kinase activity. Moreover, the prediction that the F344 specific serine residue at position 2170 generates a *de novo* consensus target sequence for multiple protein kinases suggests that the activity of the mutant allele may be altered by aberrant phosphorylation, a common carcinogenic event [43].

Interestingly, at least three previous studies reported that deletions on the long arm of human chromosome 13q12 to 13q22 were very common genetic aberrations in lymph node-negative breast cancer [44], [45], [46] **.** A recent study of Scandinavian families also suggested the presence of a third human breast cancer susceptibility locus that is distinct from *BRCA2*, on the long arm of human chromosome 13 [47]. These authors identified recurrent somatic chromosomal deletions on HSA13q21, with a minimal region of loss between 13q21-q22, although specific loss of the wild type locus was not demonstrated in these tumors. Genotyping indicated the segregation of a shared germline HSA13q21 haplotype by all affected individuals in this high-risk family. Targeted linkage analysis in 77 breast cancer families with markers from this chromosomal region provided further evidence for a putative predisposition locus (BRCA3). However, a subsequent study using 128 high-risk breast cancer families of Western European ancestry failed to corroborate these findings, and indicated that if a BRCA3 locus did exist on HSA13q, it only accounted for a small fraction of heritable, early onset breast cancers [48].

One possible explanation for the discrepancy between these two studies is the effects of gene-environment interactions. Rat mammary tumors induced by exposure to estradiol showed frequent loss of RNO12, while those induced by exposure to DMBA did not show this chromosomal loss [49], [50], indicating that gene-environment interactions can select for the loss of susceptibility loci on rat chromosome 12. If the human populations used in these studies had different environmental exposures, such gene environment interaction effects might also explain the differences observed between human mapping and somatic deletion studies. Thus, the possibility remains that the human *FRY* gene encodes a putative susceptibility gene on human Chromosome 13q, and studies are in progress to further define its role in human breast cancer.

## Supporting Information

File S1 **Contains: Table S1.** Complete list of Rat Simple Tandem Repeat (STR) Markers used for low-density linkage analysis. **Table S2.**Coverage of the genome (autosomes) by STR markers used in low-density linkage analysis. **Table S3.** Primers used to generate amplicons and sequence the *Brca2* gene from F344 and Cop rat strains. **Table S4.** Primers used to generate amplicons and sequence the Rat *Fry* gene from F344 and Cop rat strains. **Table S5.** Genbank Accession numbers for all genes sequenced in this study. **Figure S1. Histogram showing the incidence (frequency on left axis) of mammary tumors detected in N2 backcross progeny on each of the indicated days post exposure to NMU.** The incidence mammary tumors (left axis) and percent of animals with tumors (right axis) as a function of time after exposure is indicated by the green dotted line. **Figure S2.** **Schematic representation of the contig comprising overlapping Bacterial Artificial Chromosome (BACs) containing rat genomic DNA assembled using the DNA sequence of the D12Rat59 STR marker as the seed (not drawn to scale).** Rat BAC clones encompassing particular STR markers were obtained from the Children’s Hospital Oakland Research Institute (CHORI-230 BAC library) and verified by PCR amplification to contain the STR sequence. R and F refer to the forward and reverse orientation. Vertical lines indicate sequence overlap. **Figure S3.** **Metaphase chromosome spreads prepared from mouse embryo fibroblast fibroblasts isolated from (F344 X Cop)F1 progeny were hybridized with Bacterial Artificial Chromosomes containing STR markers that comprise the** ***Mcs30*** **locus or genes on RNO12 used as hybridization controls (see** **Table 2** **).** Hybridization signals are pseudo-colored red for clarity, and DAPI banding is displayed as grey values. BAC 1: CH230-152N10 containing D12Rat1 hybridized to 12q11-12. BAC 2: CH230-381M14 containing D12Rat59 hybridized to 12q11-12. BAC 3: CH230-275K13 containing D12Rat59 hybridized to 12q11-12. BAC 4: CH230-85G15O containing D12Rat59 hybridized to 12q11-12. BAC 5: CH230-151L24 containing the *Epo* gene hybridized to 12q11-12. BAC 6: CH230-123F8 containing the *PAI 1A1* gene hybridized to 12q11-12. **Figure S4**. **Relative mRNA expression levels of candidate genes from the** ***Mcs30*** **locus on Chromosome 12 in normal rat mammary tissue before and after NMU exposure.** (A). RNA was isolated from normal mammary tissue of 55 day-old female Fischer 344 and Copenhagen rats, before and 6 h, one day and 30 days after exposure exposing female F344 and Copenhagen rats at to a carcinogenic dose of NMU. Gene expression levels of *Insr* and *Stard13* on chromosome 12 were compared by semi-quantification PCR analysis. Expression levels were normalized to expression of beta-actin mRNA; (B). Image densitometry in (B) was quantified using Image J software.
(DOC)
