# Supplementary material for: Mapping of Mcs30, a New Mammary Carcinoma Susceptibility Quantitative Trait Locus (QTL30) on Rat Chromosome 12: Identification of Fry as a Candidate Mcs Gene
Source: PLoS One. 2013 Sep 2;8(9):e70930. doi: 10.1371/journal.pone.0070930 (PMC3759375; doi:10.1371/journal.pone.0070930)
Supplement: File S1 — Contains: Table S1. Complete list of Rat Simple Tandem Repeat (STR) Markers used for low-density linkage analysis. Table S2.Coverage of the genome (autosomes) by STR markers used in low-density linkage analysis. Table S3. Primers used to generate amplicons and sequence the Brca2 gene from F344 and Cop rat strains. Table S4. Primers used to generate amplicons and sequence the Rat Fry gene from F344 and Cop rat strains. Table S5. Genbank Accession numbers for all genes sequenced in this study. Figure S1. Histogram showing the incidence (frequency on left axis) of mammary tumors detected in N2 backcross progeny on each of the indicated days post exposure to NMU. The incidence mammary tumors (left axis) and percent of animals with tumors (right axis) as a function of time after exposure is indicated by the green dotted line. Figure S2. Schematic representation of the contig comprising overlapping Bacterial Artificial Chromosome (BACs) containing rat genomic DNA assembled using the DNA sequence of the D12Rat59 STR marker as the seed (not drawn to scale). Rat BAC clones encompassing particular STR markers were obtained from the Children’s Hospital Oakland Research Institute (CHORI-230 BAC library) and verified by PCR amplification to contain the STR sequence. R and F refer to the forward and reverse orientation. Vertical lines indicate sequence overlap. Figure S3. Metaphase chromosome spreads prepared from mouse embryo fibroblast fibroblasts isolated from (F344 X Cop)F1 progeny were hybridized with Bacterial Artificial Chromosomes containing STR markers that comprise the Mcs30 locus or genes on RNO12 used as hybridization controls (see Table 2 ). Hybridization signals are pseudo-colored red for clarity, and DAPI banding is displayed as grey values. BAC 1: CH230-152N10 containing D12Rat1 hybridized to 12q11-12. BAC 2: CH230-381M14 containing D12Rat59 hybridized to 12q11-12. BAC 3: CH230-275K13 containing D12Rat59 hybridized to 12q11-12. BAC 4: CH230-85G15O containing D12Ra [file pone.0070930.s001.doc]

**File S1**

**Supplementary Materials for**

**Mapping of *Mcs30,* a new mammary carcinoma susceptibility quantitative trait locus (QTL30) on rat chromosome 12: Identification of *Fry* as a candidate *Mcs* gene**

### Xuefeng Ren ^‡, 1^, Jessica C. Graham ^2, 3^, Lichen Jing ^‡^, Andrei M. Mikheev ^‡^, Yuan Gao ^‡^, Jenny Pan Lew ^‡^, Hong Xie ^‡^, Andrea S. Kim ^‡^, Xiuling Shang ^1^, Cynthia Friedman ^‡^, Graham Vail ^2^, Ming Zhu Fang ^2, 4, 5^, Yana Bromberg ^7^, and Helmut Zarbl ^2, 4, 5, 6, ‡, *^

**1** Department of Social and Preventive Medicine, The State University of New York, Buffalo, NY 14214, USA.

**2** Department of Environmental and Occupational Medicine, Robert Wood Johnson Medical School, University of Medicine and Dentistry of New Jersey Piscataway, NJ 08540, USA.

**3** Joint Graduate Program in Toxicology. Rutgers University and University of Medicine and Dentistry of New Jersey, Piscataway, New Jersey, 08854, USA.

**4** NIEHS Center for Environmental Exposures and Disease, University of Medicine and Dentistry of New Jersey and Rutgers University, Piscataway, NJ 08854 USA.

**5** Environmental and Occupational Health Sciences Institute, University of Medicine and Dentistry of New Jersey and Rutgers University, Piscataway, NJ 08854 USA.

**6** Cancer Institute of New Jersey, New Brunswick, NJ 08903-2681, USA.

**7** Department of Biochemistry and Microbiology, Rutgers University, New Brunswick, NJ 08901, USA

**‡** A portion of this work was done by XR, LJ, AM, YG, JL, HX, CF and AK in the laboratory of HZ, while he was a Member of the Fred Hutchinson Cancer Research Center (FHCRC), Seattle, WA, the NIEHS Center for Ecogenetics and Environmental Health, and the Department of Environmental and Occupational Health, University of Washington, Seattle, WA 98109 USA

* **Corresponding Author Address:**

Environmental and Occupational Health Sciences Institute, RWJMS

Rutgers, The State University of Medicine

170 Freylinghuysen Road, Room 414A, Piscataway NJ 08854, U.S.A..

**Phone: (**848) 445-2354

**FAX:** (732) 445-4161

**Email:**  [zarbl@eohsi.rutgers.edu](mailto:zarbl@eohsi.rutgers.edu)

**Key Words:** Rat *Fry* ortholog gene, NMU (N-Methyl-N-Nitrosourea), rat mammary carcinoma, genetic susceptibility, QTL mapping

This Supplemental Data file includes:

Tables S1 to S5

Figure S1 and S4

**Table S1.**

| D1RAT15 | D1RAT115 | D5RAT17 | D9RAT133 | D15RAT12 |
| --- | --- | --- | --- | --- |
| D1RAT257 | D1RAT76 | D5RAT82 | D9RAT30 | D15RAT71 |
| D1RAT266 | D1RAT81 | D5RAT13 | D9RAT18 | D16RAT8 |
| D1RAT29 | D1RAT132 | D5RAT149 | D9RAT7 | D16RAT61 |
| D1RAT32 | D2RAT10 | D5RAT95 | D9RAT100 | D16RAT34 |
| D1RAT183 | D2RAT21 | D5RAT108 | D10RAT182 | D17RAT61 |
| D1RAT38 | D2RAT161 | D5RAT35 | D10RAT73 | D17RAT15 |
| D1RAT41 | D2RAT38 | D5RAT205 | D10RAT18 | D17RAT32 |
| D1RAT272 | D2RAT54 | D6RAT68 | D10RAT8 | D18RAT53 |
| D1RAT173 | D2RAT66 | D6RAT135 | D11RAT11 | D18RAT13 |
| D1RAT236 | D3RAT17 | D6RAT21 | D11RAT34 | D19RAT15 |
| D1RAT49 | D3RAT103 | D6RAT12 | D12RAT3 | D19RAT71 |
| D1RAT277 | D3RAT167 | D7RAT31 | D12RAT35 | D20RAT4 |
| D1RAT164 | D3RAT220 | D7RAT103 | D12RAT36 | D20RAT5 |
| D1RAT282 | D3RAT15 | D7RAT86 | D13RAT70 | D20RAT10 |
| D1RAT437 | D3RAT4 | D7RAT100 | D13RAT24 | D21RAT24 |
| D1RAT198 | D4RAT115 | D7RAT11 | D13RAT32 | D21RAT111 |
| D1RAT65 | D4RAT27 | D7RAT4 | D14RAT5 | D21RAT115 |
| D1RAT290 | D4RAT40 | D8RAT51 | D14RAT11 |  |
| D1RAT22 | D4RAT6 | D8RAT46 | D14RAT39 |  |
| D1RAT181 | D4RAT200 | D8RAT104 | D14RAT94 |  |
| D1RAT293 | D4RAT68 | D8RAT65 | D15RAT5 |  |

**Table S2.**

| Chromosome | Number of Markers Used/Chromosome | Greatest Distance  Between Markers (cM) | Farthest Distance  To End of Map (cM) |
| --- | --- | --- | --- |
| 1  2  3  4  5  6  7  8  9  10  11  12  13  14  15  16  17  18  19  20 | 7  6  6  6  8  4  6  4  5  4  2  3  3  4  2  2  2  2  2  3 | 25  19  20  20  21  22  25  20  21  26  17  16  15  22  35  26  21  21  16  7 | 20  15  14  20  15  13  17  20  14  17  13  16  14  18  16  13  15  17  16  25 |

**Table S3.**

| Exon | Primer Name | Sequence | Amplicon Size |
| --- | --- | --- | --- |
| 1/2  3  4/5/6/7A  4/5/6/7B*  8/9  10A  10B*  11A  11B  11C*  11D  11E*  11F  11G*  12  13/14  15  16/17  18  19/20A  19/20B*    21    22/23/24    25/26    27A    27B* | 1/2-F  1/2-R  3-F  3-R  4/5/6/7-F  4/5/6/7-R  4/5/6/7-F  4/5/6/7-R  8/9-F  8/9-R  10-F1  10-R1  10-F2  10-R2  11A-F  11A-R  11B-F  11B-R  11C-R  11D-F  11D-R  11E-R  11F-F  11F-R  11G-R  12-F  12-R  13/14-F  13/14-R  15-F  15-R  16/17-F  16/17-R  18-F  18-R  19/20A-F  19/20A-R  19/20B-F  19/20B-R  21-F  21-R  22/23/24-F  22/23/24-R  25/26-F  25/26-R  27A-F  27A-R  27B-F  27B-R | GGC TCC ACC TCA GTC GG  GCA AGC GCT CTA CCA CT  ACT GAA AGC TGG AAT AAC AG  TAC GTA AGC CAG TCT AGC  TTG GAG ATG ACA ACT TAT GG  CTT GAA GAA GGT ATT GGA TC  CCC TGG CTG ACT ACC TC  ACA AGG GCC AGC TAT GC  TTC TTT CAG ACG TGT C 1150bp  TGT AGC AAG TCA ATT C  AGA CTT TGA GTA CAG TGG  CAG GGC TAC ACA GAA C  CAG CAT CTT GAG TCA CT  GAG CTG GTC ATA CTG TC  AGT CGG GTG CTC TTA AC  TGT ATG AGG TTC ACT TAG  CGA AGA ACA GTA TCC TAC  CTG AAG TGA CAG TCA AG  CTT GTT GTG ATC TAT CTG  AGT CTC CAA AGA GTC AC  TGG CAA GTG TCT GGT TTA C  ACT TTG TTC TCT AAG CC  CTG GAG ACT AAT GCT TC  ACT GTA GCC TAC GAG AC  AAG AGA TTG AGT ATT GC  GAA TCT TCT TGT CTA ACC C  CAA GGC ATC ATG TAT ATG  CTT TGC TTC TGA ACC TTC GT  TGC TAC ATA ATA CCC GAA G  TCT TTG AGA CCG TTC CCT C  TCA GAG CAC TTT CGC CAT C  GTT ATA CTC CGG GTC AGT GAC  TAG ATG TCC CAG GAT GGG GTG  CTC CTG ACT GCC GTG TTG  CTC CCA GCA GAA ACC ATT AG  GTG TGG TTG TGT GGT TGC TAA  CAA TGA CAG TTG GGA TCA GTT  ATC GTT CAG AGA GTG TAC C  GTG AAC AAG GCC TCT AGT T  AGT CTT CGT CTC TTC ATC GTC  TGA AAG GCT TGC TAT AAT GGC  ACA AAT CTG TCA TTC TGT TAG TGC C  GAG TTC ACT GCC AAC CTA GTC CAT A  CTC CTG AGC AAC CGC CTG AGT ATG T  CCA CGG GTT AGG TGC AGT TTA GTT C  TGG AAA GGT AAA GGA TAA ACT ATG T  TAT CTG ACA GGA TGG AGC TCT AGC A  CTA CTG ATC AGC AAA GTT ATC GAA G  TCT GTA GAG TAA ACA AAT GCG TCA A | 700bp  613bp  1583bp  1454bp  1446bp  1289bp  1326bp  1415bp  735bp  1372bp  570bp  1415bp  649bp  987bp  552bp  1252bp  1251bp  1152bp |

Note: * The primers were only used for sequencing reaction.

**Table S4.**

| Primer Set | Primer Name | Sequence | Amplicon Size |
| --- | --- | --- | --- |
| 1^st^  2^nd^  3^rd^  4^th^  5^th^  6^th^  7^th^  8^th^  9^th^  10^th^ | Fry 1^st^ -F  Fry 1^st^ -R  Fry 2^nd^ -F  Fry 2^nd^ -R  Fry 3^rd^ -F  Fry 3^rd^ -R  Fry 4^th^ -F  Fry 4^th^ -R  Fry 5^th^ -F  Fry 5^th^ -R  Fry 6^th^ -F  Fry 6^th^ -R  Fry 7^th^ -F  Fry 7^th^ -R  Fry 8^th^ -F  Fry 8^th^ -R  Fry 9^th^ -F  Fry 9^th^ -R  Fry 10^th^ -F  Fry 10^th^ -R  Fry Sequencing 1^st^-R  Fry Sequencing 2^nd^-F  Fry Sequencing 3^rd^-F | CTTCTCCAGTTGGCAACG  TTATTCTTAAGGTTGGAC  TCAAGCACGCCTTAGCAG  GGCCTGTTCATAGACTCT  AAGTCCTGTTCGGCTTCA  TCCAAGAACAACGCTCCA  CTGGAGAGCATCGAAATC  CTTCATCTCCGTACTTGG  AGAACTGGCCCGGATGTA  TGCATGGTCAGCGTCTTC  CACCTCCTCATCGCACTC  CAAGGCCATCAGGTATTC  GAAGTTTGGTGTCGTTGAC  ACACCAGGCTCGTCTGATG  TTGGGAGACGGTATGATG  CAGAGCAGGAGGTAAGCA  CCTGTGATGACGCTGAG  GGCATAAGTACCCGTCTG  CTGTGGCCCAATGACATC  TGCCTCCCGTAGTTGGT  GACGGGAAGTTCAAGTGGC  GGGCAGGCAGAATGAAG  TCTGTTTGCATTACCTTTAC | 1114bp  1205bp  1216bp  1360bp  1203bp  1119bp  1346bp  1233bp  766bp  522bp |

**Table S5.**

| **Gene Name** | **Rat Strain** | **Accession Number** |
| --- | --- | --- |
|  |  |  |
| ***Fry*** | Copenhagen | EU563851.1 |
| ***Fry*** | Fischer 344 | EU563850.1 |
|  |  |  |
| ***Brca2*** | Copenhagen | AH014113.1 |
| ***Brca2*** | Fischer 344 | AH014114.1 |
|  |  |  |
| **Stard13** | Copenhagen | KF112846 |
| **Stard13** | Fischer 344 | KF112845 |
|  |  |  |
| **Insr** | Copenhagen | KF112848 |
| **Insr** | Fischer 344 | KF112847 |


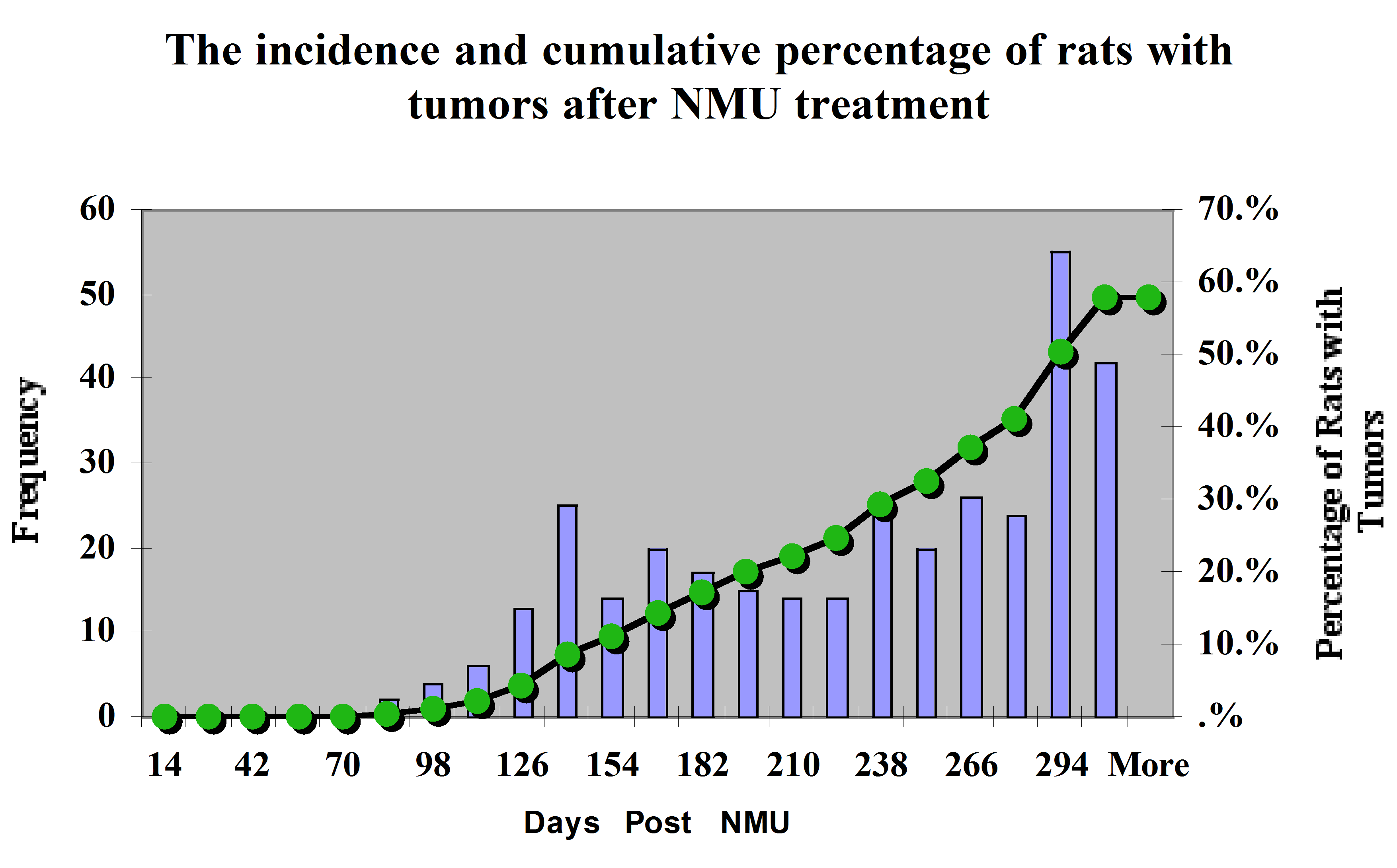


**Figure S1.**


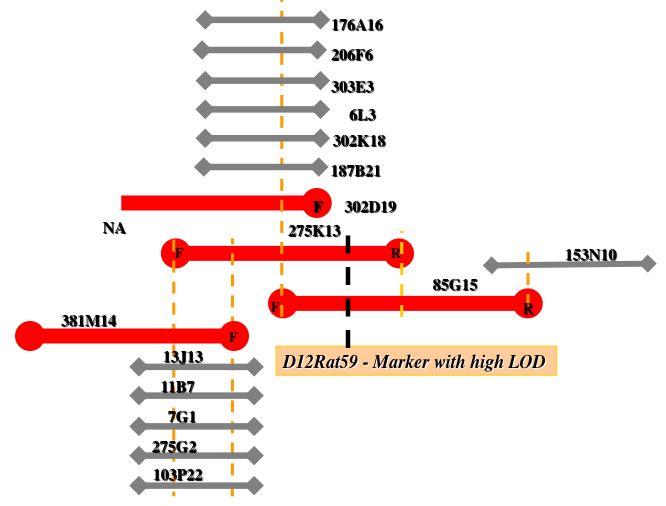


**Figure S2.**


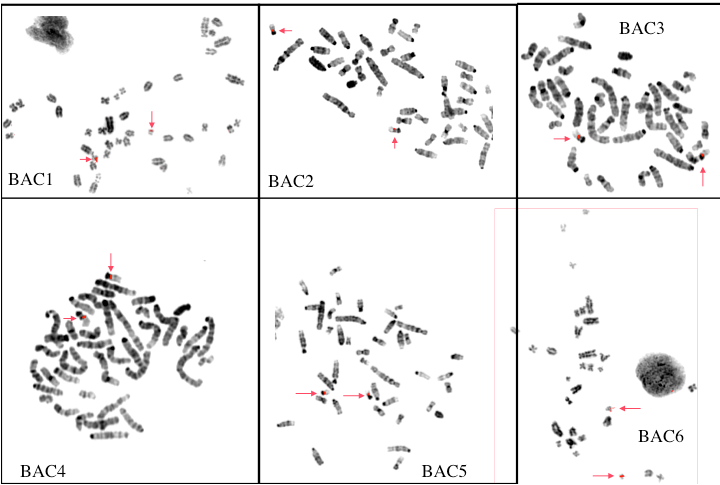


**Figure S3.**

**
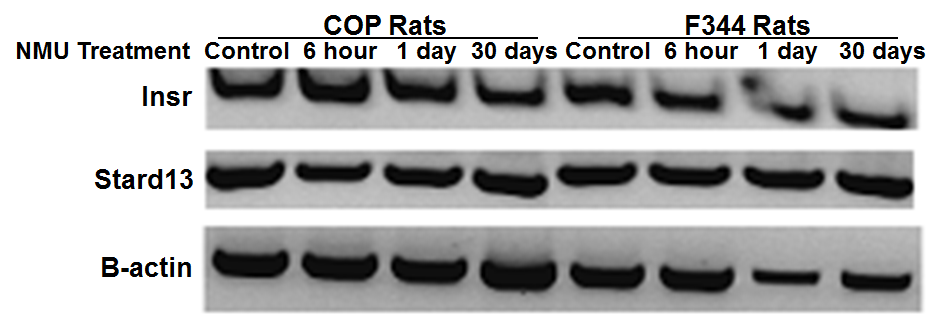
**

**A**


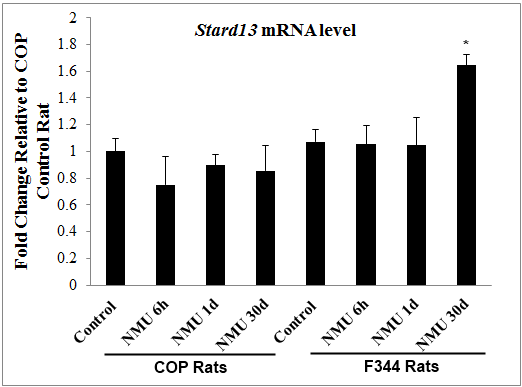

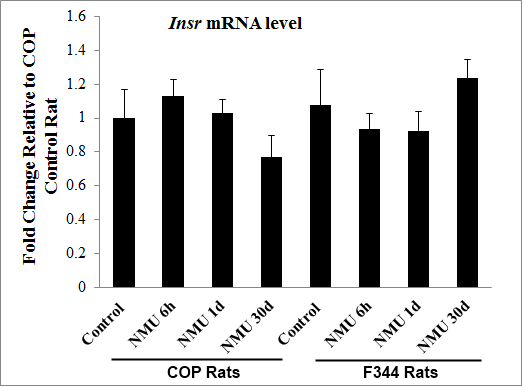
 **B**

**Figure S4.**
